# Antioxidant and Toxicity Studies of 50% Methanolic Extract of *Orthosiphon stamineus* Benth

**DOI:** 10.1155/2013/351602

**Published:** 2013-12-30

**Authors:** Mun Fei Yam, Chung Pin Lim, Lee Fung Ang, Lip Yee Por, Siew Tung Wong, Mohd. Zaini Asmawi, Rusliza Basir, Mariam Ahmad

**Affiliations:** ^1^School of Pharmaceutical Sciences, Universiti Sains Malaysia, 11800 Pulau Penang, Malaysia; ^2^Faculty of Computer Science and Information Technology, University of Malaya, 50603 Kuala Lumpur, Malaysia; ^3^International Medical University, Jalan Jalil Perkasa 19, Taman Esplanade, 57000 Kuala Lumpur, Malaysia; ^4^Faculty of Medicine and Health Sciences, Universiti Putra Malaysia, 43400 Serdang, Selangor, Malaysia

## Abstract

The present study evaluated the antioxidant activity and potential toxicity of 50% methanolic extract of *Orthosiphon stamineus* (Lamiaceae) leaves (MEOS) after acute and subchronic administration in rats. Superoxide radical scavenging, hydroxyl radical scavenging, and ferrous ion chelating methods were used to evaluate the antioxidant properties of the extract. In acute toxicity study, single dose of MEOS, 5000 mg/kg, was administered to rats by oral gavage, and the treated rats were monitored for 14 days. While in the subchronic toxicity study, MEOS was administered orally, at doses of 1250, 2500, and 5000 mg/kg/day for 28 days. From the results, MEOS showed good superoxide radical scavenging, hydroxyl radical scavenging, ferrous ion chelating, and antilipid peroxidation activities. There was no mortality detected or any signs of toxicity in acute and subchronic toxicity studies. Furthermore, there was no significant difference in bodyweight, relative organ weight, and haematological and biochemical parameters between both male and female treated rats in any doses tested. No abnormality of internal organs was observed between treatment and control groups. The oral lethal dose determined was more than 5000 mg/kg and the no-observed-adverse-effect level (NOAEL) of MEOS for both male and female rats is considered to be 5000 mg/kg per day.

## 1. Introduction

Herbal medicines have received a great deal of attention as alternative medicines in recent years in Malaysia and are sold as dietary supplements. One of the Malaysian local herbs, scientifically known as *Orthosiphon stamineus *Benth (Lamiaceae) or locally called Misai Kucing, has attracted much attention for research purposes.* Orthosiphon stamineus *is also found in other countries such as Thailand, Indonesia, and Europe. It is widely used for the treatment of many diseases, especially those affecting the urinary tract, diabetes mellitus, hypertension, rheumatism, tonsillitis, and menstrual disorders [1]. The benefits of the traditional use of *Orthosiphon stamineus* have also been supported by the isolation and identification of several possible active chemical constituents from this plant, including flavonoids [2, 3], terpenoids [4, 5], saponins, hexoses, organic acids, caffeic acid derivatives, chromene, and myo-inositol [4–6]. Among the reported compounds, the most important components of *Orthosiphon stamineus* leaves are the polyphenols; polymethoxylated flavonoids such as sinensetin and eupatorine; caffeic acid derivatives, which include rosmarinic acid, cichoric acid, and caffeic acid [6].

Notwithstanding the widespread and long time usage of this plant, little toxicological information is available regarding the safety following chronic consumption of *O. stamineus* especially the bioactive 50% methanolic extract which has been reported to be effective in protection against alcohol-induced gastropathy, CCl_4_-induced liver damage, antipyretic, anti-inflammatory, and analgesic effects [7–10]. Currently, Malaysian authorities are paying more and more attention on the safety and potential toxicity of botanicals, including medicinal plants and edible materials. Therefore, the aims of the present study were to evaluate the antioxidant and provide scientific data on the safety of* O. stamineus*, focusing on the 14-day acute and 28-day subchronic toxicity of bioactive 50% methanolic extract of *O. stamineus,* using Sprague-Dawley (SD) rats, and hence, providing guidance for selecting a safe dose of *O. stamineus *in its use as a traditional medicine.

## 2. Materials and Methods

### 2.1. Chemical

Ferrous chloride (FeCl_2_), hematoxylin, ferrozine, 2-deoxyribose, ethylenediaminetetraacetic acid (EDTA), ferrous sulfate (FeSO_4_), trichloroacetic acid (TCA), nitroblue tetrazolium (NBT), xanthine, xanthine oxidase sodium phosphate (dibasic), sodium phosphate (monobasic), butylated hydroxytoluene (BHT), butylated hydroxyanisole (BHA), manganese chloride, xylenol orange, manganese chloride, and sodium dihydrogen phosphate (NaH_2_PO_4_) were purchased from Sigma (St. Louis, MO, USA). Absolute alcohol and eosin were purchased from Riedel-de Haën (Seelze, Germany). Paraplast was purchased from Oxford Labware (St. Louis, MO, USA). Xylene was purchased from Fisher Scientific (Leics, UK). Disposable microtome blades 818 were purchased from LEICA (Germany). Thiobarbituric acid (TBA) was purchased from AppliChem (Darmstadt, Germany). 3′-hydroxy-5,6,7,4′-tetramethoxyflavone, sinensetin, and eupatorin were obtained from Indofine Chemical Company (NJ, USA). HPLC-grade acetonitrile and isopropyl alcohol were purchased from Merck (Darmstadt, Germany).

### 2.2. Plant Materials and Extract

*Orthosiphon stamineus* was grown from cuttings using standard agronomic practices at Kepala Batas, Pulau Pinang, Malaysia. The leaves of the plant were collected after flowering. The leaves were identified by Mr. Adenan Jaafar, School of Biological Sciences, Universiti Sains Malaysia. A voucher specimen (number 10810) was deposited at the Herbarium of School of Biological Sciences, Universiti Sains Malaysia. The leaves were rinsed and dried in oven at 40°C for 2 days. The dried leaves were then ground by an electric grinder to a coarse powder and weighed. Subsequently, 300 g of powdered leaves of *O. stamineus* was extracted with 5 L methanol : water (50 : 50 v/v) at 60°C (for 8 hours) by maceration method. The resulting 50% methanolic extract of *O. stamineus* (MEOS) was concentrated using a Büchi-RE121 evaporator (Büchi Laboratorium-Technik, Switzerland) equipped with a Büchi-B169 vacuum system and then lyophilized in a Hetovac VR-1 (HETO Lab Equipment, Denmark) freeze dryer. The weight of the MEOS was recorded and the final plant-to-extract ratio was about 6%. The MEOS was then kept in desiccators in a refrigerator (0–4°C). The MEOS was freshly prepared daily by dissolving in distilled water.

### 2.3. HPLC Study

#### 2.3.1. HPLC Instrumentation

HPLC analysis was performed using a Shimadzu-LC system (Shimadzu, Japan) equipped with a CBM-20A controller, LC-20AT pump, DGU-20A5 degasser, SIL-20A autosampler, SPD-20AV detector, and CTO-10ASvp column oven.

#### 2.3.2. Chromatographic Conditions

Chromatographic separations were achieved using an Agilent Eclipse Plus C18 (250 × 4.6 mm i.d.; 5 *μ*m). A Zorbax guard fittings kit packed with replaceable Eclipse Plus C18 Guard column (12.5 × 4.6 mm i.d.; 5 *μ*m) was used to protect the analytical column. A reverse-phase HPLC assay was carried out using an isocratic system with a flow rate of 1 mL/min, a column temperature of 25°C, and a mobile phase of acetonitrile : isopropyl alcohol : 20 mM phosphate buffer (NaH_2_PO_4_) (30 : 15 : 55 v/v), with pH adjusted to 3.5 using 85% phosphoric acid. The UV detection was set at 340 nm. The injection volume was 20 *μ*L of solution. Total run time was less than 20 min for each injection. Data was acquired and processed with LC-Solution software. The peaks were detected at 340 nm and identified using reference standards of 3′-hydroxy-5,6,7,4′-tetramethoxyflavone, sinensetin, and eupatorin. Sinensetin and eupatorin were selected as standards because these two compounds were found to be the bioactive ingredients in the plant [11–13].

### 2.4. Antioxidant

#### 2.4.1. Ferrous Ion Chelating Activity

Ferrous chelating activity was determined according to the method of Dinis et al. [14]. MEOS was added to a solution of 0.05 mL ferrous chloride (FeCl_2_) (2 mM). The reaction was initiated by the addition of 0.2 mL ferrozine (5 mM) and the mixture was shaken vigorously and left standing at room temperature (24 ± 2°C) for 10 min. Absorbance of the solution was then measured spectrophotometrically (U-2000, Hitachi, Japan) at wavelength 562 nm. The percentage of inhibition of ferrozine-Fe^2+^ complex formation was calculated using the following formula:

(1) $$\begin{array}{c} \text{Ferrous}\;\text{ion}\;\text{chelating}\;\text{activity}=\left[\frac{\left(A_{0}-A_{1}\right)}{A_{0}}\right]\times 100, \end{array}$$

where *A*_0_ was the absorbance of the control and *A*_1_ was the absorbance in the presence of extracts. The experiment was run together with butylated hydroxytoluene (BHT) and butylated hydroxyanisole (BHA) as positive controls.

#### 2.4.2. Hydroxyl Radical Scavenging Activity

Competition between deoxyribose and the extract against hydroxyl radical generated from the Fe^3+^/ascorbate/ethylenediaminetetraacetic acid (EDTA)/hydrogen peroxide (H_2_O_2_) system was measured to determine the hydroxyl radical scavenging activity of MEOS [15]. The reaction mixture consisted of 0.30 mL of 0.02 M sodium phosphate buffer (pH 7.0), 0.15 mL of 10 mM 2-deoxyribose, 0.15 mL of 10 mM FeSO_4_, 0.15 mL of 10 mM EDTA, 0.15 mL of 10 mM H_2_O_2_, 0.525 mL of H_2_O, and 0.075 mL of extract. The reaction mixture was incubated at 37°C for 2 h and the formed TBARS were measured. Seventy five milliliter of 2.8% trichloroacetic acid and 0.75 mL of 1.0% TBA in 50 mM NaOH were added to test tubes and boiled for 20 min. After cooling the mixture, absorbance was measured at 520 nm. The percentage of inhibition of inhibition rate of 2-deoxyribose oxidation by hydroxyl radical was given in the following formula:

(2) $$\begin{array}{c} \text{Hydroxyl}\;\text{radical}\;\text{scavenging}\;\text{activity}=\left[\frac{\left(A_{0}-A_{1}\right)}{A_{0}}\right]\times 100, \end{array}$$

where *A*_0_ was the absorbance of the control and *A*_1_ was the absorbance in the presence of extracts.

#### 2.4.3. Superoxide Anion Radical Scavenging Activity

Superoxide anion radical generated by the xanthine/xanthine oxidase system was determined spectrophotometrically by monitoring the product of nitroblue tetrazolium (NBT) [16]. The reaction mixture consisted of 1.0 mL of 0.05 M phosphate buffer (pH 7.4), 0.04 mL of 3 mM xanthine, 0.04 mL of 3 mM EDTA, 0.04 mL of 0.15% bovine serum albumin, 0.04 mL of 15 mM NBT, and 0.04 mL of sample solution. After incubation at 25°C for 10 min, the reaction was initiated by adding 0.04 mL of 1.5 U/mL xanthine oxidase and carried out at 25°C for 20 min. After 20 min, the absorbance of the reaction mixture was measured at 560 nm. Decreased absorbance of the reaction mixture indicates increased superoxide anion radical scavenging activity.

#### 2.4.4. Lipid Peroxidation Inhibition Study

Fresh livers were obtained from healthy male adult SD rats. They were then homogenized according to the method [17] for the preparation of 40% (w/v) liver homogenates in methanol. 80 *μ*L of liver homogenate was mixed with 10 *μ*L of Fenton's reagent (containing 5 *μ*L of 5 mM manganese chloride and 5 *μ*L of 50 mM hydrogen peroxide) and 10 *μ*L of various concentrations of MEOS (for control, the extract was replaced with distilled water). The mixture was incubated for 30 min at 37°C to produce lipid peroxidation. The mixture was then added with 900 mL FOX reagent (containing 49 mg ammonium ferrous sulfate in 50 mL of 250 mM H_2_SO_4_, 0.397 g of BHT, and 0.038 g of xylenol orange in 950 mL methanol). Absorbance of the mixture was read at 560 nm after 30 min of incubation at room temperature. The percentage of inhibition was calculated with the following equation:

(3) $$\begin{array}{c} \%\;\text{of}\;\text{inhibition}=\left[\frac{\left(A_{0}-A_{1}\right)}{A_{1}}\right]\times 100, \end{array}$$

where *A*_0_ was the absorbance in the presence of extracts and *A*_1_ was the absorbance of the control.

### 2.5. Toxicology Studies

#### 2.5.1. Experimental Animals

Eight-week-old male and female Sprague-Dawley rats (160–190 g) were purchased from the Animal House, School of Pharmaceutical Sciences, Universiti Sains Malaysia. The rats were acclimatised to laboratory conditions for 7 days prior to performing the experiments. All rats were kept at 26 ± 3°C, with a light/dark cycle of 12 hours. The rats were housed in a single polycarbonate cage (3 rats per cage) with free access to food (normal laboratory chow, Gold Coin) and tap water *ad libitum*. All the procedures were performed according to the Animal Ethics Guidelines of Universiti Sains Malaysia.

#### 2.5.2. Acute Toxicity Study

The experiment was performed according to the Organisation for Economic Cooperation and Development (OECD) revised up and down procedure for acute toxicity testing [18]. All the rats were fasted overnight and weighed before the extract was administered. A maximum dose of 5000 mg/kg of MEOS was administered by oral gavage to 5 healthy female adult Sprague-Dawley rats. The administration volume was adjusted between 1 and 2 mL for each and every rat. After administration of MEOS to the first rat, the rat was observed for clinical signs of toxicity for the first hour, then hourly for three hours, and then periodically throughout 48 hours. Other rats were administrated sequentially at 48-hour intervals if the first rat survived after 48 hours of treatment. All the experimental rats were maintained under close observation throughout the 14 days and the number of mortality was recorded. The LD_50_ was predicted to be above 5000 mg/kg if three or more rats survived after the experimental period [19].

#### 2.5.3. Subchronic Toxicity Study

Experimental Sprague-Dawley rats of either sex were randomly assigned to 4 groups (*n* = 12; six males and six females per group), and their weights were recorded. Different doses of MEOS (1250, 2500, or 5000 mg/kg) were prepared in distilled water and administered daily as single doses to different groups of rats: group 1 (5000 mg/kg), group 2 (2500 mg/kg), and group 3 (1250 mg/kg), while group 4 (control) received only distilled water. Toxic manifestations and mortality were monitored daily for 28 days. The bodyweights of all the rats were measured and recorded at the end of every week and the treated rats were anesthetised using CO_2_ after 28 days of treatment. Blood samples were collected via cardiac puncture and transferred into nonheparinised and EDTA-containing tubes for both biochemical and haematological analyses, respectively [20]. Thereafter, the rats were killed by cervical dislocation. All organs, brain, heart, lungs, thymus, liver, kidneys, adrenal glands, sex organs (ovaries and uterus for female rats; testes for male rats), spleen, stomach, and gut (begining from small intestine until the end of large intestine), of all experimental rats were excised, weighed, and examined macroscopically. Vital organs such as lungs, kidneys, livers, and stomach were then preserved in 10% formalin for histopathological study [19].

#### 2.5.4. Relative Organ Weight

The excised organs were weighed individually. The relative organ weight index of each organ to its bodyweight was calculated as (weight of organ/bodyweight of rats on the day of sacrifice) × 100% [20].

#### 2.5.5. Blood Analyses

Haematological and biochemical analyses were performed at the Pathology Laboratory, Lam Wah Ee Hospital, Penang. Complete blood cell counts were determined using a fully automated haematological analyser Abbott Cell-Dyn 3500 (Abbott Laboratories, IL, USA), while serum biochemistry tests were performed using a COBAS Integra 800 (Roche, Germany) [20].

#### 2.5.6. Histopathological Study

Vital organs (kidney and liver) were processed using a Citadel 1000 Histokinette (Shandon Scientific Ltd., Cheshire, UK). These tissues were embedded in paraffin using a Histo-Center II-N (Barnstead/Thermolyne Corp., Dubuque, IA, USA) and cut into 5 *μ*m thick sections with a Reichert-Jung Histocut 820 II (Cambridge Instrument GmbH, Nussloch, Germany). All the sections were stained with haematoxylin and eosin and examined microscopically [20].

### 2.6. Statistical Analysis

Statistical analysis was carried out using the Statistical Package for Social Sciences (SPSS). All the data are indicated as mean ± standard error of mean (SEM) and were analysed using one-way analysis of variance (ANOVA). Significant differences between the groups were determined using a Dunnett-comparison test with *P* < 0.05 taken as significant.

## 3. Results

### 3.1. HPLC Analysis

The objective of HPLC analysis in this experiment was to standardize the MEOS using sinensetin, eupatorin, and 3′-hydroxy-5,6,7,4′-tetramethoxyflavone. From the results, the percentages of sinensetin, eupatorin, and 3′-hydroxy-5,6,7,4′-tetramethoxyflavone determined in MEOS were 1.12 ± 0.026%, 0.94 ± 0.025%, and 0.46 ± 0.016% (w/w), respectively.

### 3.2. Antioxidant Activities

#### 3.2.1. Ferrous Ion Chelating Activity

**Figure 1:**
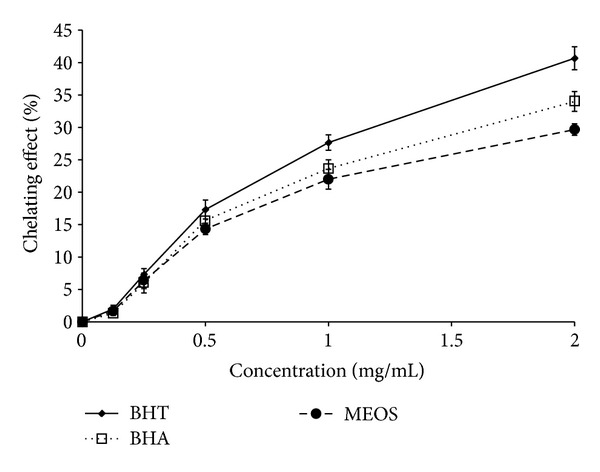
Ferrous ion chelating activity of MEOS (*n* = 3). Data are expressed as mean ± SD.

Ferrozine can quantitatively form the complexes with Fe^2+^. Chelating activity of the MEOS with ferrous ion was 1.6% at 0.125 mg/mL. In addition, there was an increase of chelating activity to 30% at 2 mg/mL which was slightly lower than positive control (BHA and BHT) (Figure 1).

#### 3.2.2. Hydroxyl Radical Scavenging Activity

**Figure 2:**
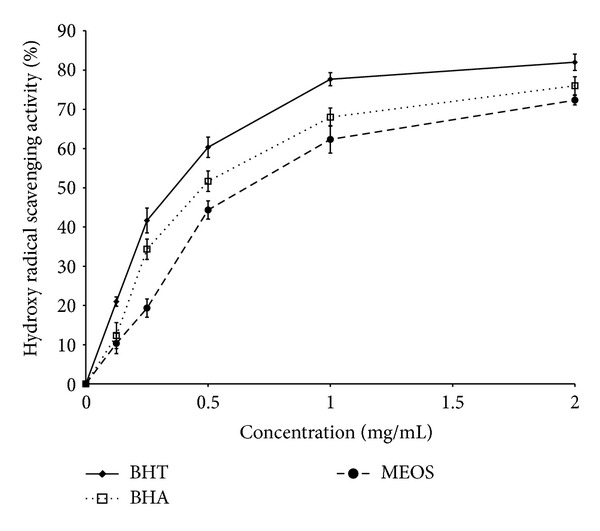
Hydroxyl radical scavenging activity of MEOS (*n* = 3). Data are expressed as mean ± SD.

The hydroxyl radical scavenging activity was investigated by using the Fenton reaction. As shown in Figure 2, addition of less than 2 mg/mL of MEOS effectively inhibited the formation of hydroxyl radicals linearly up to 72%.

#### 3.2.3. Superoxide Anion Radical Scavenging Activity

**Figure 3:**
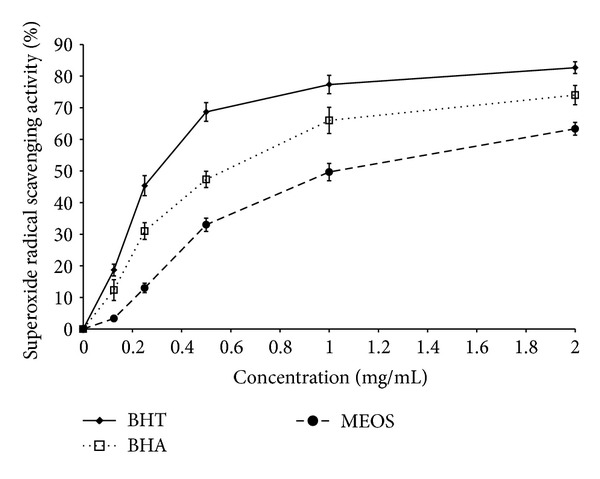
Superoxide radical scavenging activity of MEOS (*n* = 3). Data are expressed as mean ± SD.

Superoxide anion radical scavenging activity of MEOS was determined by the xanthine oxidase system.  Figure 3 showed the percentage inhibition of superoxide radical generated by BHT, BHA, and MEOS. The inhibition activity of MEOS reached 63% when 2 mg/mL of extract was used.

#### 3.2.4. Lipid Peroxidation Inhibition Study

Antilipid peroxidation activity of MEOS was determined by the FOX method. IC_50_ of liver homogenate lipid peroxidation of MEOS was 0.34 ± 0.024 mg/mL.

### 3.3. Toxicology Studies

#### 3.3.1. Acute Toxicity Study

In this experiment, oral administration of 5000 mg/kg of MEOS did not cause any visible signs of toxicity to the rats. No changes were observed in the behavior and mortality of the animals over the 14 days. All five female SD rats survived until the end of the experiment with no mortality were recorded. The LD_50_ determined was greater than 5000 mg/kg.

#### 3.3.2. Subchronic Toxicity Study

**Figure 4:**
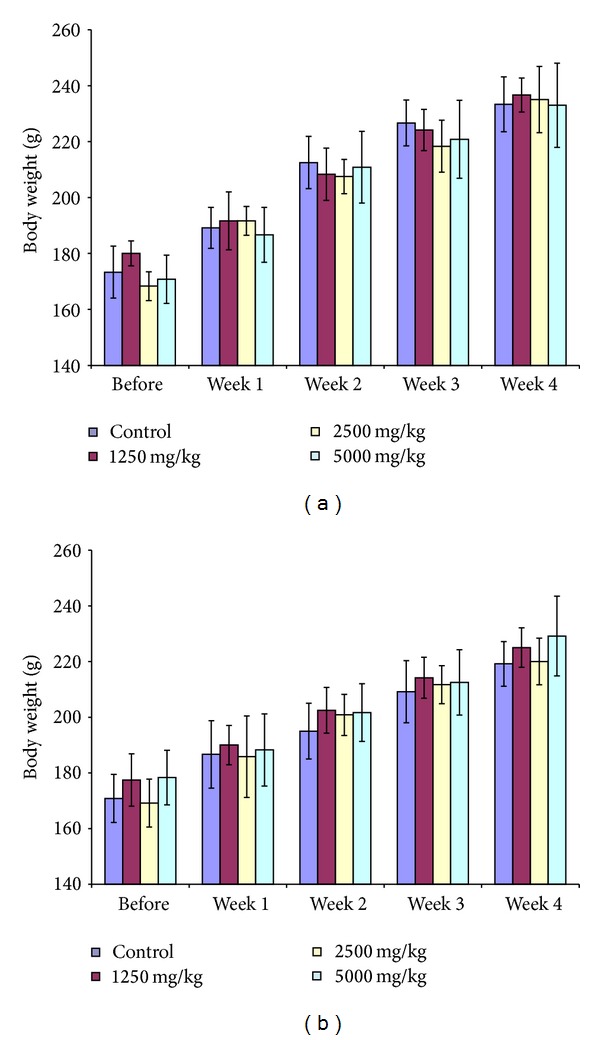
The effect of daily oral administration of MEOS on the bodyweight of (a) male and (b) female rats (*n* = 6). Data are expressed as mean ± SEM.

In subchronic toxicity study, there were no observable changes in the general behaviour of all the treated rats (groups 1, 2, and 3) as compared to the control group. No significant changes were detected in either the bodyweights (Figure 4) or relative organ weights (Table 1) of all the treated rats. In addition, no death was recorded in both sexes of the treatment groups as well as their respective control group, during or after the course of the experiment. The controls and the treated rats appeared uniformly healthy at the end of the experiment and throughout the 28 days treatment period.

#### 3.3.3. Blood Analyses

There were no significant differences observed in any of the haematological parameters tested (red blood cell count (RBC), haemoglobin concentration (Hgb), haematocrit (Ht), mean corpuscular volume (MCV), mean corpuscular haemoglobin (MCH), mean corpuscular haemoglobin concentration (MCHC), total white blood cell count (WBC), or white blood cell differential count) in any of the treated rats as compared to control rats (Table 2). Meanwhile, analyses of biochemical parameters (alanine transaminase (ALT), aspartate transaminase (AST), alkaline phosphatase (ALP), creatinine, bilirubin, urea, sodium, potassium, and chlorine) also showed no significant differences in any of the parameters tested between the control and the treated groups of both sexes (Table 3).

#### 3.3.4. Histopathological Study

No lesions or pathological changes were observed in the organs of either sex of the MEOS treated rats as compared to their respective control groups.

## 4. Discussion

*Orthosiphon stamineus* Benth. (Lamiaceae) is an important medicinal plant in Malaysia. It has been widely used for treatment of various ailments. Many studies which involved both *in vivo *and *in vitro *experiments have reported that the *Orthosiphon stamineus* leaves possess a wide range of pharmacological properties such as, anti-inflammatory, analgesic, gastroprotective, antipyretic, and hepatoprotective effects. The present study was conducted to determine the antioxidant potential, which is responsible for the wide range of pharmacological properties of this plant and to investigate the possible harmful effects of MEOS in experimental animals at the specific doses chosen, since the safety use of the plant is of concern to the Malaysian authorities. The toxicity of MEOS in rats was evaluated by both acute 14-day and subchronic 28-day toxicological studies. Methanol was chosen as our solvent since many literature reviews showed that this extract was very effective in many pharmacological activities such as antipyretic, anti-inflammatory, and analgesic effects as well as a good protective agent against alcohol-induced gastropathy and CCl_4_-induced liver damage [7–10].

Antioxidant capacity is one of the commonly used parameters to determine the bioactive components which exhibited pharmacological activities. *Orthosiphon stamineus* has been shown to be a potent scavenger of a variety of reactive radical species such as DPPH and ABTS [7]. Therefore, in the present study, the antioxidant potential of MEOS was tested by Ferrous ion chelating activity, hydroxyl radical scavenging activity, and superoxide anion radical scavenging activity. Our study showed that the antioxidant activity of MEOS was comparable to the positive controls: BHA and BHT. In the antioxidant activities, we reported that MEOS can strongly reduce the hydroxyl and supercritical onion radicals. This finding is comparable to the previous study which reported that MEOS inhibited lipid peroxidation in different animal models [7, 10]. In addition, it has been long reported that there is a strong association between the termination of free radical propagation in biological systems with the reduction of chronic diseases, DNA damage, mutagenesis, carcinogenesis, and inhibition of pathogenic bacterial growth [21]. Hence, as a potent antioxidant, it is not surprising that *Orthosiphon stamineus* is effective in treating various oxidative stress-related chronic diseases such as diabetes, alcohol-induced stomach ulcer, and CCl_4_-induced liver damage.

In acute 14-day toxicity study, a limited dose was selected and performed on the experimental rats. Its purpose was to determine a proper range of doses to be used in the subsequent subchronic 28-day toxicological study [19]. In this experiment, a single dose of 5000 mg/kg MEOS was given orally to five female Sprague-Dawley rats. No significant mortality or alteration in the behaviour patterns of the treated rats was observed compared to their respective controls.

During the subchronic 28-day toxicity study, the rats were treated orally with different doses of MEOS (1250, 2500, and 5000 mg/kg/day) for 28 days. The results obtained were comparable to those in the acute toxicity study. Both control and treated rats of both sexes appeared generally healthy during and throughout the experimental period. No mortality was recorded and no toxicity signs were detected in any of the treated rats. Both evaluation of the acute and subchronic toxicity of MEOS had indicated that a single oral administration of MEOS up to 5000 mg/kg dose caused neither visible signs of toxicity nor mortality to experimental rats. Hence, the LD_50_ of MEOS determined is more than 5000 mg/kg.

Generally, an increase or decrease in bodyweight of an animal has been used as an indicator of adverse effect of drugs and chemicals [22]. In our study, all the treated animals survived beyond the observation period. The bodyweight of both control and treatment groups increased gradually throughout the experimental period with no significant difference. The increase in bodyweight was not significantly different as compared to the control group, in both acute and subchronic toxicity studies. All the rats at each dosage level continued to gain weight throughout the experimental period, suggesting that growth inhibition did not occur during this course of repeated dosage.

In addition to bodyweight, relative organ weight has also been used as another basic indicator to determine whether the rats have been exposed to harmful agents [19, 22]. Their organs will tend to swell or damage if they were subjected to toxic substances. This will subsequently alter their organ-to-bodyweight ratios as compared to the respective controls. In the present study, the relative organ weights of liver, lungs, spleen, sex organs, heart, and kidneys in all the treated male and female rats were not significantly different (*P* < 0.05) from those of the control groups.

Analysis of blood parameters in animal studies could be very useful when evaluating the risk of human toxicity as the changes in the haematological system provide a predictive value for toxicity [18]. These blood parameters are often called haematological parameters. Several important haematological parameters were selected in this study to evaluate the toxicity of MEOS. From the results, the haematological profile of all the treated rats showed no significant difference with the control group (Table 2). This indicated that the MEOS did not affect the haematopoiesis system of the treated rats.

Several important biochemical parameters were also included in this toxicity study. These biological parameters are indicators for organ toxicity. For example, kidney functions were evaluated by means of serum urea and creatinine. Increase of blood creatinine has been shown to be a good indicator of negative impact in kidney functions [23]. While the increase levels of AST and ALT in the blood are associated with damage of hepatic cells [24, 25]. The results of the experiment suggested that the kidney and liver functions were not altered for both the treated male and female rats. There were no statistically significant differences in creatinine, AST, and ALT levels between controls and treated animals at any dosage levels. Hence, these findings suggest that MEOS does not cause any hepatotoxicity and kidney damage to the rats. Both these organs were selected because kidneys and liver are the first organs to show toxicity when the rats are exposed to potential toxic substances. Based on the statistical analyses of these biochemical parameters, no significant difference was observed in any of the parameters tested for either the treated or normal rats (Table 3).

Histopathological studies were conducted on vital organs of all the rats. According to Wang et al., impaired organs often have abnormal atrophy [26]. However, from our results, no related histopathological changes were observed. Gross examination in necropsy and microscopic examination revealed no changes that might be due to the administration of MEOS (data not shown). No significant changes or damages were observed in the morphology of all the isolated vital organs, either from the rats treated with MEOS or control rats (data not shown). The isolated organs showed normal architecture. These results again suggested that daily repeated oral administration of MEOS did not cause any detrimental changes or morphological disturbances to the rats or to their vital organs. The no-observed-adverse-effect level (NOAEL) of MEOS determined was 5000 mg/kg bodyweight/day (>3000 mg/kg bodyweight/day) and considered to be a non-hazard compound.

## 5. Conclusion

This finding is comparable with Mohamed et al.'s [19] and Abdullah et al.'s (2009) in which *O*. *stamineus* alcoholic extract did not show any toxic effect in rats during both acute and subchronic studies. Daily oral administration of 50% methanolic extract of *O. stamineus* (MEOS) at doses of 1250, 2500, and 5000 mg/kg to both male and female Sprague-Dawley rats for 28 days did not result in mortality and was not associated with adverse effects as reflected in the observation of general condition, growth, body and organ weights and hematological and biochemical values. Thus, its oral lethal dose for both male and female rats is in excess of 5000 mg/kg. There were no abnormalities in necropsy and histopathological findings. An NOAEL from the present study was determined to be 5000 mg/kg per day for rats under the condition tested. Further investigation on the preclinical and clinical studies of the extract will be necessary to determine a safe dose before it becomes a potential drug and to protect the population from possible toxic effects of MEOS. It would be interesting to assess the toxic effect of the compounds found in MEOS such as eupatorin and sinensetin.

## Figures and Tables

**Table:** (a)

% organ weight/bodyweight	Treatment for 28 days			
	Control	50% methanolic extract of *Orthosiphon stamineus *		
		1250 mg/kg	2500 mg/kg	5000 mg/kg
Male				
Brain	0.42 ± 0.02	0.39 ± 0.01	0.43 ± 0.02	0.42 ± 0.02
Heart	1.54 ± 0.03	1.44 ± 0.13	1.43 ± 0.08	1.37 ± 0.06
Liver	2.34 ± 0.10	2.42 ± 0.16	2.48 ± 0.15	2.46 ± 0.14
Thymus	0.14 ± 0.01	0.16 ± 0.01	0.15 ± 0.01	0.15 ± 0.01
Spleen	0.27 ± 0.02	0.27 ± 0.01	0.25 ± 0.01	0.27 ± 0.02
Kidney (right)	0.35 ± 0.01	0.37 ± 0.02	0.33 ± 0.01	0.35 ± 0.01
Kidney (left)	0.32 ± 0.01	0.33 ± 0.01	0.32 ± 0.01	0.33 ± 0.01
Adrenal gland	0.01 ± 0.00	0.02 ± 0.00	0.01 ± 0.00	0.01 ± 0.00
Lungs	0.67 ± 0.05	0.71 ± 0.06	0.63 ± 0.02	0.63 ± 0.03
Testis (right)	0.56 ± 0.02	0.54 ± 0.01	0.55 ± 0.01	0.55 ± 0.02
Testis (left)	0.54 ± 0.03	0.55 ± 0.01	0.54 ± 0.02	0.56 ± 0.01
Stomach	2.74 ± 0.31	2.63 ± 0.06	2.93 ± 0.08	2.56 ± 0.11
Stomach (empty)	0.57 ± 0.01	0.59 ± 0.01	0.51 ± 0.02	0.54 ± 0.02
Gut	6.64 ± 0.21	6.38 ± 0.14	6.45 ± 0.14	6.32 ± 0.30
Gut (empty)	3.27 ± 0.03	3.44 ± 0.11	3.17 ± 0.04	3.24 ± 0.13

Data are expressed as mean ± SEM.

**Table:** (b)

% organ weight/bodyweight	Treatment for 28 days			
	Control	50% methanolic extract of *Orthosiphon stamineus *		
		1250 mg/kg	2500 mg/kg	5000 mg/kg
Female				
Brain	0.40 ± 0.00	0.39 ± 0.00	0.40 ± 0.01	0.38 ± 0.01
Heart	1.70 ± 0.03	1.65 ± 0.02	1.68 ± 0.02	1.60 ± 0.04
Liver	2.61 ± 0.08	2.69 ± 0.09	2.81 ± 0.04	2.66 ± 0.06
Thymus	0.11 ± 0.01	0.09 ± 0.01	0.09 ± 0.00	0.10 ± 0.01
Spleen	0.22 ± 0.01	0.25 ± 0.02	0.23 ± 0.02	0.24 ± 0.01
Kidney (right)	0.28 ± 0.01	0.27 ± 0.01	0.28 ± 0.01	0.26 ± 0.01
Kidney (left)	0.27 ± 0.00	0.27 ± 0.01	0.28 ± 0.00	0.26 ± 0.01
Adrenal gland	0.02 ± 0.00	0.02 ± 0.00	0.02 ± 0.00	0.02 ± 0.00
Lungs	0.66 ± 0.01	0.63 ± 0.02	0.65 ± 0.02	0.67 ± 0.03
Ovaries	0.13 ± 0.01	0.09 ± 0.01	0.10 ± 0.01	0.11 ± 0.01
Uterus	0.21 ± 0.00	0.16 ± 0.01	0.19 ± 0.02	0.15 ± 0.01
Stomach	2.74 ± 0.18	2.71 ± 0.14	2.71 ± 0.06	2.69 ± 0.09
Stomach (empty)	0.58 ± 0.01	0.53 ± 0.01	0.51 ± 0.01	0.50 ± 0.01
Gut	7.29 ± 0.25	6.82 ± 0.21	6.80 ± 0.11	6.94 ± 0.30
Gut (empty)	4.06 ± 0.08	3.83 ± 0.11	3.63 ± 0.13	3.82 ± 0.15

Data are expressed as mean ± SEM.

**Table:** (a)

	Unit	Treatment for 28 days			
		Control	50% methanolic extract of *Orthosiphon stamineus *		
			1250 mg/kg	2500 mg/kg	5000 mg/kg
Male					
White blood cell count	10^9^/L	9.57 ± 0.32	11.05 ± 0.48	10.78 ± 0.59	10.73 ± 0.21
Neutrophils	10^9^/L	2.46 ± 0.18	3.09 ± 0.22	3.15 ± 0.31	3.18 ± 0.43
Lymphocytes	10^9^/L	6.67 ± 0.24	6.94 ± 0.46	6.72 ± 0.29	6.67 ± 0.41
Monocytes	10^9^/L	0.24 ± 0.03	0.62 ± 0.09	0.53 ± 0.15	0.54 ± 0.05
Eosinophils	10^9^/L	0.02 ± 0.00	0.04 ± 0.01	0.05 ± 0.01	0.05 ± 0.01
Basophils	10^9^/L	0.18 ± 0.03	0.35 ± 0.04	0.33 ± 0.08	0.30 ± 0.02
Red blood cell count	10^12^/L	8.55 ± 0.12	8.36 ± 0.17	8.43 ± 0.20	8.24 ± 0.03
Hemoglobin	g/L	14.80 ± 0.21	14.76 ± 0.14	15.02 ± 0.32	14.49 ± 0.08
Hematocrit	%	0.75 ± 0.01	0.74 ± 0.02	0.75 ± 0.02	0.71 ± 0.00
Mean red blood cell volume	fL	88.03 ± 0.74	88.15 ± 1.27	88.51 ± 1.74	86.47 ± 0.81
Mean corpuscular Hb	pg	17.34 ± 0.19	17.70 ± 0.31	17.75 ± 0.22	17.58 ± 0.07
Mean corpuscular Hb concentration	g/L	19.71 ± 0.25	20.16 ± 0.40	20.12 ± 0.40	20.39 ± 0.16
Platelets	10^9^/L	1205.61 ± 26.53	1149.19 ± 29.80	1145.28 ± 67.30	1125.36 ± 18.04
Mean platelet cell volume	fL	8.12 ± 0.12	8.46 ± 0.17	8.26 ± 0.19	8.11 ± 0.13

Data are expressed as mean ± SEM.

**Table:** (b)

	Unit	Treatment for 28 days			
		Control	50% methanolic extract of *Orthosiphon stamineus *		
			1250 mg/kg	2500 mg/kg	5000 mg/kg
Female					
White blood cell count	10^9^/L	7.66 ± 0.96	6.77 ± 0.43	6.31 ± 0.72	7.24 ± 0.35
Neutrophils	10^9^/L	1.69 ± 0.32	1.63 ± 0.18	1.47 ± 0.16	1.78 ± 0.18
Lymphocytes	10^9^/L	5.35 ± 0.68	4.62 ± 0.52	4.37 ± 0.55	4.26 ± 0.95
Monocytes	10^9^/L	0.34 ± 0.08	0.30 ± 0.09	0.31 ± 0.05	0.30 ± 0.03
Eosinophils	10^9^/L	0.02 ± 0.01	0.02 ± 0.00	0.02 ± 0.00	0.01 ± 0.00
Basophils	10^9^/L	0.27 ± 0.07	0.20 ± 0.04	0.22 ± 0.04	0.19 ± 0.02
Red blood cell count	10^12^/L	7.76 ± 0.17	7.79 ± 0.06	7.25 ± 0.36	7.48 ± 0.19
Hemoglobin	g/L	14.16 ± 0.14	14.44 ± 0.12	13.56 ± 0.29	13.64 ± 0.50
Hematocrit	%	0.71 ± 0.01	0.71 ± 0.00	0.67 ± 0.02	0.68 ± 0.01
Mean red blood cell volume	fL	89.47 ± 1.64	90.93 ± 0.52	87.80 ± 1.95	91.09 ± 1.20
Mean corpuscular Hb	pg	18.30 ± 0.25	18.54 ± 0.14	18.01 ± 0.23	18.35 ± 0.56
Mean corpuscular Hb concentration	g/L	20.14 ± 0.18	20.41 ± 0.18	19.85 ± 0.23	20.16 ± 0.56
Platelets	10^9^/L	1054.14 ± 57.95	1021.22 ± 14.30	987.46 ± 21.65	1035.83 ± 55.79
Mean platelet cell volume	fL	7.99 ± 0.10	7.78 ± 0.19	7.23 ± 036	7.55 ± 0.10

Data are expressed as mean ± SEM.

**Table 3:** Biochemical values of male and female rats treated with 50% methanolic extract of *Orthosiphon stamineus* for 28 days.

	Unit	Treatment for 28 days			
		Control	50% methanolic extract of *Orthosiphon stamineus *		
			1250 mg/kg	2500 mg/kg	5000 mg/kg
Male					
Aspartate transaminase (AST)	U/L	122.74 ± 2.19	131.94 ± 3.53	126.79 ± 9.40	121.81 ± 2.45
Alanine aminotransferase (ALT)	U/L	64.51 ± 2.98	62.43 ± 2.59	64.50 ± 3.86	65.22 ± 2.73
Urea	U/L	6.31 ± 0.34	6.65 ± 0.34	6.50 ± 0.40	6.74 ± 0.14
Creatinine	*μ*mol/L	40.56 ± 0.81	39.59 ± 0.30	41.08 ± 1.16	39.33 ± 0.45
Alkaline phosphatase (ALP)	*μ*mol/L	300.56 ± 7.18	309.63 ± 10.08	309.50 ± 11.49	316.86 ± 13.42
Bilirubin	mmol/L	1.80 ± 0.00	1.80 ± 0.00	1.80 ± 0.00	1.80 ± 0.00
Sodium	g/L	142.53 ± 0.36	142.53 ± 0.22	141.96 ± 0.58	142.22 ± 0.31
Potassium	g/L	4.69 ± 0.12	4.64 ± 0.06	4.72 ± 0.07	4.65 ± 0.08
Chlorine	g/L	102.72 ± 0.60	102.71 ± 0.33	103.75 ± 0.28	103.53 ± 0.61
Female					
Aspartate transaminase (AST)	U/L	127.83 ± 2.51	127.72 ± 4.98	122.61 ± 10.41	117.28 ± 8.10
Alanine aminotransferase (ALT)	U/L	54.33 ± 2.32	52.17 ± 3.32	53.78 ± 3.41	52.94 ± 1.72
Urea	U/L	7.83 ± 0.46	7.39 ± 0.08	8.00 ± 0.39	7.19 ± 0.29
Creatinine	*μ*mol/L	50.83 ± 2.41	47.72 ± 1.11	47.11 ± 1.27	44.50 ± 0.25
Alkaline phosphatase (ALP)	*μ*mol/L	247.50 ± 8.33	249.72 ± 21.56	250.22 ± 21.23	267.33 ± 9.95
Bilirubin	mmol/L	1.80 ± 0.00	1.80 ± 0.00	1.80 ± 0.00	1.80 ± 0.00
Sodium	g/L	142.83 ± 0.60	142.94 ± 0.20	141.44 ± 0.20	141.11 ± 0.26
Potassium	g/L	4.23 ± 0.14	4.19 ± 0.15	4.04 ± 0.11	4.14 ± 0.15
Chlorine	g/L	102.50 ± 0.62	103.39 ± 0.54	103.17 ± 0.51	103.83 ± 0.46

Data are expressed as mean ± SEM.
